# Alternative activation of macrophages by prostacyclin synthase ameliorates alcohol induced liver injury

**DOI:** 10.1038/s41374-021-00531-7

**Published:** 2021-06-10

**Authors:** Xue-yin Pan, Ling Wang, Hong-mei You, Miao Cheng, Yang Yang, Cheng Huang, Jun Li

**Affiliations:** 1 Inflammation and Immune Mediated Diseases Laboratory of Anhui Province, Anhui Institute of Innovative Drugs, School of Pharmacy, Anhui Medical University, Hefei, 230032 China; 2 The key Laboratory of Anti-inflammatory of Immune Medicines, Ministry of Education, Hefei, 230032 China; 3 Institute for Liver Diseases of Anhui Medical University, Hefei, China

**Keywords:** miRNAs, Gene silencing

## Abstract

Alcoholic liver disease (ALD) is a major cause of chronic liver disease worldwide. Macrophages exhibit different functional states and are classified as classically activated (M1) and alternatively activated (M2) macrophages. However, the mechanisms that govern M1/M2 polarization in chronic ALD remain to be elucidated. Prostacyclin (PGI_2_) synthase (PTGIS) is an enzyme of the prostaglandin pathway which catalyzes the conversion of Prostaglandin H_2_ (PGH_2_) to PGI_2_. PTGIS has anti-inflammatory properties. However, the function of PTGIS in ALD has not yet been determined. In this study, we demonstrated that PTGIS was downregulated in ALD and forced PTGIS expression in vivo using recombinant adeno-associated viral vector-packed PTGIS overexpression plasmid, which alleviated the inflammatory response and suppressed the macrophage M1 phenotype in mice. Loss- and gain-of function-experiments demonstrated that forced PTGIS expression inhibited the macrophage switch to the M1 phenotype and promoted M2 polarization. Furthermore, we identified the genes regulated by PTGIS through RNA-sequencing (RNA-seq) analysis. Gene ontology and KEGG pathway analyses showed that PTGIS regulates many genes involved in the immune response and is enriched in the Janus kinase/signal transducers and activators of transcription (JAK/STAT) signal transduction pathway, which plays an important role in regulating macrophage polarization. The proteins interacting with JAKs were predicted using the STRING database. The overlap between the RNA-seq and the STRING database was interleukin-6; this indicated that it was involved in macrophage polarization regulated by JAK/STAT signaling. We further explored the microRNAs that could regulate the expression of PTGIS through TargetScan. The results of luciferase assay illustrated that the expression of PTGIS was regulated by miR-140-3p.1. These results imply that PTGIS plays a pivotal role in ALD, partly by influencing macrophage polarization.

## Abstract (web summary)

Prostacyclin synthase (PTGIS) levels are decreased by elevated miR-140-3p.1 expression in chronic alcoholic liver disease. Inducing PTGIS expression alleviates chronic alcohol-induced liver injury by promoting macrophage M2 polarization and inhibiting the M1 phenotype through the JAK/STAT pathway. Interestingly, inducing PTGIS expression also increases IL-6 expression, which has not yet been explained.

## Introduction

Alcohol consumption is a leading risk factor for global disease burden and causes substantial health loss [1]. Alcoholic liver disease (ALD) is the leading cause of liver-related disorders and contributes to 25% of alcohol-related deaths worldwide [2]. ALD is characterized by progressive liver steatosis, fibrosis, and cirrhosis. Consumption of alcohol increases gut permeability, which leads to the translocation of both microbes and microbial products, including lipopolysaccharide (LPS), β-glucan, and other pathogen-associated molecular patterns, into the portal circulation, activating innate immune responses in the liver [3, 4]. Macrophages are highly heterogeneous, plastic populations that undergo pleiotropic coordinated responses to tissue damage through distinct programs of activation, known as classical activation (M1) or alternative activation (M2) [5, 6]. M1 activation is mainly induced by Th1 cytokines, such as interferon-gamma (IFNγ), or bacterial molecular patterns, including endotoxin/LPS.

The macrophage switch to an M1 phenotype plays a key role in releasing pro-inflammatory enzymes and cytokines, such as inducible nitric oxide synthase (iNOS), interleukin (IL)-1β, and tumor necrosis factor (TNF)-α [7–9]. In contrast, Th2 cytokines, such as IL-4 and IL-13, drive macrophages to switch to the M2 phenotype, which promotes the resolution of inflammation and is involved in tissue repair and remodeling [5, 6]. Stimulation of the IFNγ receptor triggers Janus kinase (JAK)-mediated tyrosine phosphorylation and subsequent dimerization of (STAT)1 [10]. IL-4Rα signals through a JAK-STAT6 pathway [11], and many of the genes associated with mouse M2 macrophages are regulated by STAT6, including arginase (Arg) 1, macrophage mannose receptor 1 (also known as CD206), resistin-like-α (also known as Fizz1), and chitinase 3-like 3 (also known as Ym1) [12]. In addition, PI3K and PPARγ play an important role in M2 polarization [13, 14].

The polarization phenotype of macrophages in response to ethanol is complex, with increased expression of both M1 and M2 polarization markers as well as that of both pro- and anti-inflammatory mediators [15]. Louvet et al. showed that activation of cannabinoid receptor 2 expressed in Kupffer cells inhibits M1 polarization and favors M2 response, thereby exerting anti-steatogenic roles by paracrine interactions with hepatocytes [15]. Limiting M1 macrophage polarization attenuates alcohol-induced liver injury, and IL-10 released from M2 polarized macrophages promotes M1 apoptosis [9]. Interestingly, ethanol-induced M1 polarization is modifiable; M1 macrophages can be shifted to an M2 phenotype [16]. However, little is currently known regarding the mechanisms of hyperpolarization and the impact of these complex phenotypes on the development of chronic ethanol-induced inflammation in the liver.

MicroRNAs (miRNAs), endogenous small non-coding RNAs, can inhibit target gene expression by directly binding to complementary sequences in the 3′ untranslated region (3′UTR) of mRNAs and thus are involved in many physiological processes, including cellular differentiation and immune cell activation [17]. miRNAs may play pivotal roles in modulating macrophage activation, polarization, tissue infiltration, and resolution of inflammation [18–20].

Prostaglandins are key lipid mediators that regulate physiological functions and inflammatory responses in health and disease [21]. They are synthesized from arachidonic acid via cyclooxygenases (1 and 2) to generate unstable prostaglandin H_2_ (PGH_2_), which is further processed by terminal synthases into the major prostaglandins (PGI_2_, PGE_2_, PGD_2_, and PGF2α) or thromboxane A2 [21]. The PTGS pathway is frequently dysregulated in cancers [22]. PGI_2_ synthase (PTGIS), also known as PGIS or CYP8A1, is a member of family 8 (CYP8) in the cytochrome P450 superfamily. PTGIS catalyzes the conversion of PGH_2_ to PGI_2_ [23]. Altered PTGIS plays an important role in preventing tumor growth and progression [24–27]. In addition, PTGIS plays an anti-inflammatory role in colorectal cancer [28], and prostaglandin G/H synthase 2 involved in the initial response to LPS stimulation [29]. However, whether PTGIS is involved in regulating macrophage polarization in ALD has not yet been determined. Therefore, in this study, we investigated whether PTGIS is involved in regulating macrophage polarization in ALD.

## Materials and methods

### Murine model of ALD

Six-to-eight-week-old male C57BL/6j mice (18–22 g weight) were purchased from the Laboratory Animal Center of Anhui Medical University. All animal procedures were reviewed and approved by the Institutional Animal Experimental Ethics Committee. Mice were housed in a temperature-controlled room (22 °C). After a week of adaptive breeding, mice were randomly divided into control diet (CD) and EtOH-fed groups (eight mice per group).

A mouse model of chronic plus single binge ethanol gavage was used as previously described [30]. For chronic alcohol administration, in the first 5 days, all mice were fed a liquid control diet. Thereafter, EtOH-fed mice were fed a liquid diet containing 5% v/v ethanol for 10 days, whereas the CD-fed mice were fed isocaloric maltose-dextrin. On day 16, EtOH-fed mice were administrated a single dose of ethanol gavage (5 g/kg body weight, 20% ethanol), while CD-fed mice were administrated an isocaloric dose of dextrin maltose gavage. Nine hours after gavage, the mice were euthanized, and blood and liver tissue samples were collected for further analysis.

### Isolation of primary macrophages

Liver macrophages were isolated following a previously established method [31]. Briefly, a 20-G catheter was inserted through the mouse portal vein, and the inferior vena cava was cut. The liver was perfused with PB (40 mL of PBC diluted in 1 L ddH_2_O). The PBC solution preparation method was as follows: NaCl (103.75 g), KCl (6.25 g), and Hepes (28.70 g) were dissolved in 350 mL H_2_O; 1 M NaOH (75 mL) was added, and ddH_2_O was subsequently added to make up a volume of 500 mL. Next, the liver was perfused with digestion buffer (type IV collagenase [35 mg], pronase[35 mg], 1 M CaCl_2_ solution [1 mL], PB solution [99 mL]). After digestion, the liver was disrupted in 1% bovine serum albumin (BSA) solution. Single cells were passed through a 200-mesh sieve cell strainer and fractionated using 25 and 50% Percoll (Sigma-Aldrich, St. Louis, USA). The interface fraction was washed and adhered to plastic in medium. Macrophages adhered to the plastic, and the nonadherent fraction was washed off with Dulbecco’s Modified Eagle’s Medium (DMEM, Hyclone, China) with 15% fetal bovine serum (FBS; Biological Industries, Israel). The non-adherent cells were removed, and the remaining adhered cells were considered macrophages. Macrophages from three mice were pooled for protein and RNA isolation, because of the limited number of macrophages available from each animal.

### Cell culture and treatment

RAW264.7 cells were cultured in DMEM containing 10% FBS at 37 °C in a humidified atmosphere containing 5% CO_2_.

### Blood measurements

The enzyme activities of alanine aminotransferase (ALT, C009-2-1), and aspartate aminotransferase (AST, C010-2-1), as well as triglyceride (TG, A-110-1-1) and total cholesterol (T-CHO, F002-1-1) levels were detected using commercial assay kits according to the manufacturers’ introductions (Nanjing Jiancheng Bioengineering Institute, Nanjing, China). The absorbance values at 510 nm were obtained with a micro-plate reader model 680 (Bio-Rad Laboratories, Hercules, CA, USA).

### Histopathology

The specimen was sequentially fixed in 10% formalin for 24–48 h and embedded in paraffin for histopathological analysis. Thin sections (5 μM) were stained with hematoxylin and eosin staining (H&E) for histopathological analysis. The stained tissues were scanned using Panoramic MIDI (3D HISTECH, Hungary) and viewed using CaseViewer slice software.

### Oil-Red O staining

Fresh liver tissues were immersed in freezing medium and stored at −80 °C. The liver tissues were sliced, washed with PBS three times (5 min), and stained with Oil Red O reagent according to the manufacturer’s protocol (Nanjing Jiancheng Bioengineering Institute, Nanjing, China). The stained tissues were scanned using Panoramic MIDI (3D HISTECH, Hungary) and viewed using CaseViewer slice software.

### Immunohistochemistry (IHC)

IHC was performed on paraffin sections to examine the expression of F4/80 and PTGIS using a microwave-based antigen retrieval technique. Sections were incubated with rabbit anti-F4/80 (1:50 dilution) and anti-PTGIS (1:200 dilution) overnight at 4 °C. After incubation with the secondary antibody and 3, 30-diaminobenzidine tetrahydrochloride, the slides were counterstained with hematoxylin. Non-immune rabbit IgG instead of the primary antibody was used for the negative control (NC). The stained tissues were examined with an inverted fluorescence microscope (OLYMPUS IX83, Tokyo, Japan) or scanned using Panoramic MIDI (3D HISTECH, Hungary) and viewed using CaseViewer slice software.

### Transfection of PTGIS overexpression plasmid constructs and PTGIS siRNA into RAW264.7 cells

Overexpression or knockdown of PTGIS was assessed through western blotting and real-time quantitative PCR (RT-qPCR) analysis after transfection of RAW264.7 cells with PTGIS overexpression plasmids or siRNA. All siRNAs and plasmid constructs were obtained from GenePharma Co., Ltd. Cells (3 ×10^5^/mL) were seeded in six-well plates and transfected with the PTGIS plasmid or siRNA and control constructs were mixed with lipofectamine 2000 transfection reagent (Invitrogen, Carlsbad, CA, USA) according to the manufacturer’s protocol. Cells were incubated with Opti-MEM at 37 °C and 5% CO_2_ for 6 h. Subsequently, cells were cultured in DMEM containing 10% FBS. The PTGIS overexpressing or knockdown cells were treated with LPS (1 μg/mL) and IFNγ (10 ng/mL) for 24 h to polarize the M1 phenotype, or were cultured with IL-4 (15 ng/mL) for 24 h to induce M2 macrophages. The cells were subsequently harvested for western blotting, RT-qPCR, or other analyses.

### ELISA assay

The concentrations of IL-1β, MCP-1, IL-10, and TGF-β in the serum and culture supernatant were determined using Joyee mouse ELISA kit (Joyee Biotechnics Co.,Ltd, Anhui, China) according to the manufacturer’s introductions. Optical density values were measured at 450 nm. Three independent experiments were performed, and each sample was quantified with three replicates.

### Immunofluorescence

To determine the expression of PTGIS in vivo, immunofluorescence staining was performed. Briefly, freshly dissected liver tissues were embedded in opti-mum cutting temperature compound, and the sections (8 μm thick) were cut with a cryotome Cryostat (Leica, CM 1950, Germany). Frozen tissue sections were incubated with 10% BSA for 1 h at room temperature (RT) to block nonspecific staining. After rinsing, the slides were incubated with primary antibodies for PTGIS (1:200 dilution) overnight at 4 °C. The slides were then washed twice with PBS (5 min) and incubated with FITC (green)-conjugated secondary antibody (1:50 dilution) for 1.5 h at RT in the dark.

RAW264.7 cells were fixed and permeabilized in 4% paraformaldehyde and 0.2% TritonX-100 in PBS for 10 min. Nonspecific binding was blocked with 10% BSA for 1 h at RT. Subsequently, the cells were incubated with primary antibodies for PTGIS (1:200 dilution) overnight at 4 °C. Sections were washed twice with PBS and incubated with fluorescein-labeled secondary antibody at a dilution of 1:50 for 1.5 h at RT in the dark. Slides were mounted in mounting media with DAPI for 5 min at RT. After washing twice with PBS, the slides were covered with DABCO and the stained tissues were examined with an inverted fluorescence microscope (OLYMPUS IX83, Tokyo, Japan). Fluorescence density was measured using Image Pro Plus 6.

### Western blotting analysis

Total proteins from cultured RAW264.7 cells and primary macrophages cells were extracted with radioimmunoprecipitation lysis buffer (containing 1% phenylmethylsulfonyl fluoride; Beyotime, China). The concentration of protein was determined using a bicinchoninic acid protein assay kit (Beyotime, Jiangsu, China). Proteins of each sample (30–50 μg) were separated via sodium dodecyl sulfate-polyacrylamide gel electrophoresis (10%) and transferred onto polyvinylidene fluoride (PVDF) membranes (Millipore Corp, Billerica, MA, USA). The membranes were blocked in 5% skim milk for 3 h to block nonspecific binding. Subsequently, membranes were incubated with the primary antibodies against PTGIS (Abcam, ab23668, 1:800), STAT1 (Bioworld, P42224, 1:800), p-STAT1 (Bioworld, AP0246P, 1:800), STAT6 (Bioss, bsm-52239R, 1:800), p-STAT6 (Bioworld, BS4181, 1:800) and β-actin overnight at 4 °C, followed by incubation with secondary antibody at RT for 1 h. Signals were captured with the ChemiDoc^TM^ Image System (Bio-Rad, USA). The intensities of the western blotting bands were quantified and analyzed using the ImageJ software (NIH, Bethesda, MD, USA).

### RNA extraction and RT-qPCR analysis

Total RNA was extracted from primary macrophages isolated from a mouse model, and cultured RAW264.7 cells were using TRIzol (Invitrogen, USA) according to the manufacturer’s instructions. The total RNA was uniformly quantified to 500 ng/μL using a Thermo Scientific NanoDrop 2000 Spectrophotometer (Thermo Scientific, USA). Single-stranded complementary DNA was synthesized from total RNA using the PrimeScript® RT reagent kit (TaKaRa, Japan) according to the manufacturer’s instructions. SYBR-Green RT-qPCR analysis was performed for *Ptgis*, *Il-1β*, *Tnf-α*, *Il-6*, i*NO2*, *Il-10*, *Tgf-β*, *Arg-1*, *Cd163*, and *Gapdh* mRNAs using SYBR Green Master Mix (TaKaRa, Japan) and the CFX Connect^TM^ Real-Time System (Bio-Rad, USA). The primers used in this study were as follows: mus *Ptgis*: Forward: 5′- TCCTCAAGAATCCGGAAGCC-3′, Reverse: 5′-TCTTCTGTGGGAGTGTGGTC-3′; mus *Il-1β*: Forward: 5′-GAAGAAGAGCCCATCCTCTG-3′, Reverse: 5′-TCATCTCGGAGCCTGTAGTG-3′; mus *Tnf-α*: Forward: 5′-GACAGTGACCTGGACTGTGG-3′, Reverse: 5′-TGAGACAGAGGCAACCTGAC-3′; mus *Il-6*: Forward: 5′-AGTCCGGAGAGGAGACTTCA-3′, Reverse: 5′-ATTTCCACGATTTCCCAGAG-3′; mus i*Nos*: Forward: 5′-CCTTGTTCAGCTACGCCTTC-3′, Reverse: 5′-CTTCAGAGTCTGCCCATTGC-3′; mus *Il-10*: Forward: 5′-TGCACTACCAAAGCCACAAG-3′, Reverse: 5′-TCAGTAAGAGCAGGCAGCAT-3′; mus *Tgf-β*: Forward: 5′-TTGCTTCAGCTCCACAGAGA-3′, Reverse: 5′-CAGAAGTTGGCATGGTAGCC-3′; mus *Arg-1*: Forward: 5′-GCAGTTGGAAGCATCTCTGG-3′, Reverse: 5′-GAGAAAGGACACAGGTTGCC-3′; mus *Cd163*: Forward: 5′-ATGGGTGGACACAGAATGGT-3′, Reverse: 5′-AGCTCACAGCCACAACAAAG-3′; mus *Gapdh*: Forward: 5′-GGGTCCCAGCTTAGGTTCAT-3′, Reverse: 5′-CCAATACGGCCAAATCCGTT-3′. The ratio for the mRNA of interest index was normalized to *Gapdh* and expressed as the mean ± s.e.m.

### Recombinant adeno-associated virus-mediated PTGIS overexpression in mice

Mouse PTGIS over-expression plasmid labeled with green fluorescent protein (GFP) was obtained from Hanbio Biotechnology Co., Ltd. (Shanghai, China). The PTGIS plasmid was packaged with recombinant adeno-associated-virus for overexpression of PTGIS in vivo. Male C57BL/6j mice (age, six-to-eight weeks, weight, 18-22 g) were housed at the Animal Experimental Center for one week to adapt to the environment. The tails of mice were wiped with alcohol to expand the tail vein for injection. Mice were slowly injected with 100 μL of recombinant adeno-associated-virus-packaged PTGIS over-expression plasmid at a concentration of 1 × 10^11^ v.g/mL/mouse through the tail vein using a 0.5 mL insulin syringe. One week later, mice were fed either control diet or EtOH diet.

### RNA sequencing (RNA-seq) and computational analysis

Briefly, RAW264.7 cells transfected with pEX3 and pEX3-PTGIS plasmid were collected and lysed with TRIzol reagent (Invitrogen, USA) according to the manufacturer’s instructions. RNA was sequenced using the Illumina HisSeq 1000 at Novogene in Beijing (https://en.novogene.com). Raw data were analyzed using Illumina Casava 1.8, and three types of sequence reads—reads with adapter, no exact base information, or low-quality reads—were filtered and removed. RNA (3 μg) was used for library preparation with the NEBNext® UltraTM RNA Library Prep Kit for Illumina® (NEB, USA). The reference genome and gene model annotation files were downloaded directly from the genome website. The index of the reference genome was built using Hisat2 v2.0.5, and paired-end clean reads were aligned to the reference genome using Hisat2 v2.0.5. Differential expression analysis of two conditions/groups (two biological replicates per condition) was performed using the DESeq2 R package (1.16.1). The threshold we used to screen upregulated or downregulated mRNAs was |log_2_ fold-change | > 1 and *p* value < 0.05.

### Bioinformatic analyses

The potential miRNA binding sites of PTGIS were obtained using the TargetScan 7.1 software (http://www.targetscan.org/). The protein interactions with PTGIS, JAK1, JAK2, and JAK3 were predicted using the online STRING database (https://string-db.org/).

### Dual-luciferase reporter assay

The binding site of 3′-UTR-PTGIS and miRNA-140-3p.1 was detected via the dual-luciferase reporter assay. Briefly, the wt-PTGIS and mut-PTGIS sequences were inserted into the KpnI and HindIII sites of the pSI-Check2 promoter vector (Hanbio Biotechnology Co., Ltd., Shanghai, China) in a dual-luciferase reporter assay. Finally, the 293 T cells were grown in 96-well plates until they reached 50–70% confluence. Subsequently, 0.16 μg PTGIS plasmid was co-transfected with mmu-miR-140-3p.1 mimics/NC. The dual-luciferase assay was carried out 48 h after transfection. The experiment was independently performed three times.

### Statistical analysis

Data are expressed as the mean ± s.e.m. One-way analysis of variance and the Newman–Keuls post hoc test (Prism 8.3 GraphPad Software, Inc, San Diego, CA, USA) were used to analyze the results. *p* < 0.05 was considered statistically significant.

## Results

### Alcohol consumption causes liver injury, steatosis, and inflammation

**Fig. 1:**
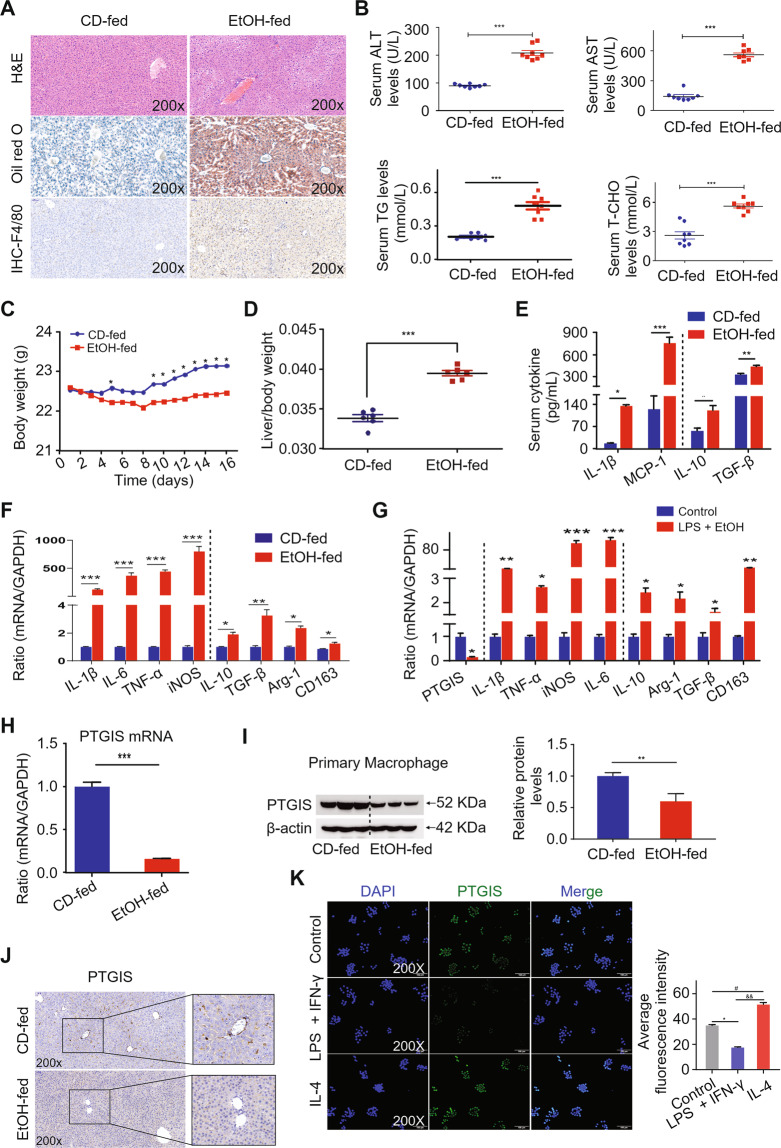
PTGIS expression and macrophage polarization in vivo and in vitro. **A** Representative pictures of H&E, Oil Red O and F4/80 staining of liver tissue sections were shown. **B** Serum ALT, AST, TG, and T-CHO levels were measured. **C** Body weight were shown. **D** The ratio of liver-to-body weight. **E** Serum cytokines were detected by ELISA. **F** The mRNA levels of M1 markers and M2 markers in primary macrophages isolated from liver tissues were detected by RT-qPCR analysis. **G** PTGIS mRNA expression and M1, M2 markers in vitro were measured by RT-qPCR analysis. The mRNA (**H**) and protein levels (**I**) of PTGIS in primary macrophages were measured by RT-qPCR and western blotting analysis. **J** Representative pictures of PTGIS immune staining were shown. **K** Protein level of PTGIS in vitro was detected by immunofluorescence. Values represent the mean ± s.e.m for 3–4 independent experiments. ^*^p < 0.05, ^**^p < 0.01, ^***^p < 0.001 as indicated.

Mice with chronic ethanol feeding were characterized by liver injury. To investigate the degree of liver injury induced by ethanol consumption, we performed H&E staining and Oil Red O staining, measured serum ALT, AST, TG, and T-CHO levels, and determined the body weights and liver-to-body weight ratios. Histopathological analysis showed extensive steatosis, liver cell cord derangement, and intercellular space dilatation in EtOH-fed mice (Fig. 1A). Serum ALT, AST, TG, and T-CHO levels were substantially elevated in the EtOH-fed mouse group (Fig. 1B). As shown in Fig. 1C, body weight decreased in the early stage and subsequently increased in the EtOH-fed group. However, the liver-to-body weight ratio was elevated in EtOH-fed group (Fig. 1D). We further investigated macrophages infiltration in liver tissues using IHC analysis. Intriguingly, chronic alcohol feeding caused a marked elevation in the total number of macrophages in C57BL/6j mice, as assessed via F4/80 immunostaining (Fig. 1A), associated with an obvious increase in M1-polarized macrophages and a moderate rise of M2-polarized macrophages (Fig. 1E and F). The in vitro results were consistent with the in vivo results (Fig. 1G). The results indicate that chronic alcohol consumption leads to liver injury, steatosis, and significant macrophage M1 polarization in vivo and in vitro.

### Chronic alcohol consumption decreases PTGIS expression both in vivo and in vitro

To identify the expression changes of PTGIS induced by chronic alcohol consumption, western blotting, RT-qPCR, and IHC analyses were performed. As shown in Fig. 1H–J, PTGIS expression significantly decreased in the EtOH-fed group. As shown in Fig. 1G, PTGIS mRNA expression was significantly decreased in RAW264.7 cells cultured with EtOH and LPS. The immunosignals of PTGIS in EtOH-fed mice substantially decreased (Fig. 1J). The immunofluorescence signal of PTGIS significantly decreased in M1-polarized RAW264.7 cells and increased in M2-polarized RAW264.7 cells (Fig. 1K). These results indicate that chronic alcohol consumption decreased PTGIS expression both in vivo and in vitro.

### Recombinant adeno-associated virus-mediated overexpression of PTGIS protects against chronic alcohol consumption-induced liver injury, steatosis, and inflammation in vivo

**Fig. 2:**
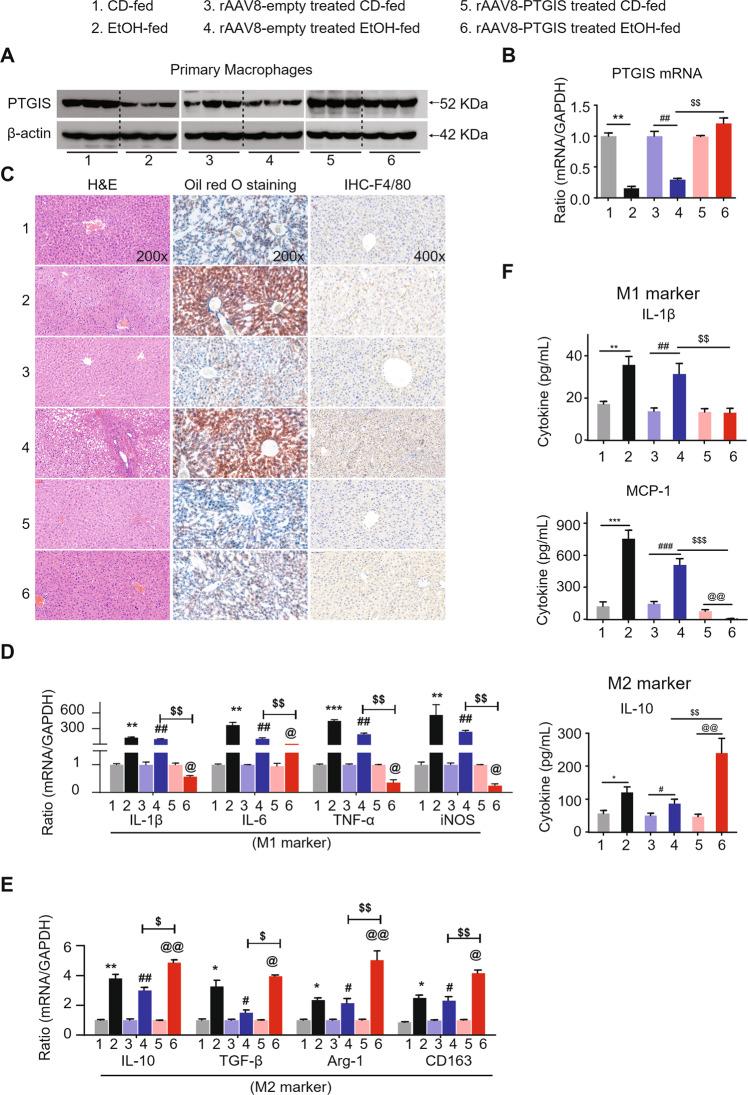
Liver-specific PTGIS overexpression alleviates alcohol-induced liver injury and inhibits primary macrophage M1 polarization. **A** PTGIS protein expression in primary macrophages were determined by western blotting analysis. **B** The mRNA levels of PTGIS in primary macrophages was measured by RT-qPCR analysis. **C** Representative pictures of H&E, Oil Red O staining, and F4/80 staining of liver tissue sections were shown (Original magnification was as indicated). **D**, **E** The mRMA expression of M1 and M2 marker were detected. **F** Serum cytokines of M1 and M2 marker were measured by ELISA. Values represent the mean ± s.e.m. ^*^p < 0.05, ^**^p < 0.01, ^***^p < 0.001, ^#^*p* < 0.05, ^##^*p* < 0.01, ^###^*p* < 0.001, ^*$*^*p* < 0.05, ^$$^*p* < 0.01, ^$$*$*^*p* < 0.001, ^@^*p* < 0.05^, @*@*^*p* < 0.01 as indicated.

The expression of PTGIS, both at protein and mRNA levels, was significantly downregulated by EtOH consumption both in vivo and in vitro. We investigated whether PTGIS-targeted therapy could prevent chronic alcohol-induced liver injury in vivo. The eGFP signal in the liver transfected with the PTGIS overexpression plasmid indicated a successful infection of the virus (Fig. S1A). PTGIS expression was substantially increased in primary macrophages isolated from recombinant adeno-associated viral vector (rAAV8)-PTGIS-transfected liver compared to rAAV8-empty-vector-treated liver tissue at both the protein and mRNA levels (Fig. 2A, B). As shown in Fig. 2C, overexpression of PTGIS in liver tissues alleviated alcohol-induced liver injury (H&E staining) and inhibited liver steatosis (Oil Red O staining) and macrophage infiltration (F4/80 staining) compared to empty-virus-treated mice in the EtOH-fed group. Forced expression of PTGIS dramatically elevated the body-weight and suppressed the liver-to-body weight ratio of mice compared to the empty-virus-infected group in the EtOH-fed group (Fig. S1B, S1C). As shown in Fig. 2D, E, forced PTGIS expression in vivo could inhibit M1 polarization and promote macrophage switch to the M2 phenotype. The ELISA results were consistent with those of the RT-qPCR analysis (Fig. 2F). These results indicated that forced expression of PTGIS in vivo inhibited M1 polarization and promoted M2 polarization and may be a potential therapeutic target for chronic alcohol-induced liver injury.

### The expression of PTGIS is regulated by miR-140-3p.1

**Fig. 3:**
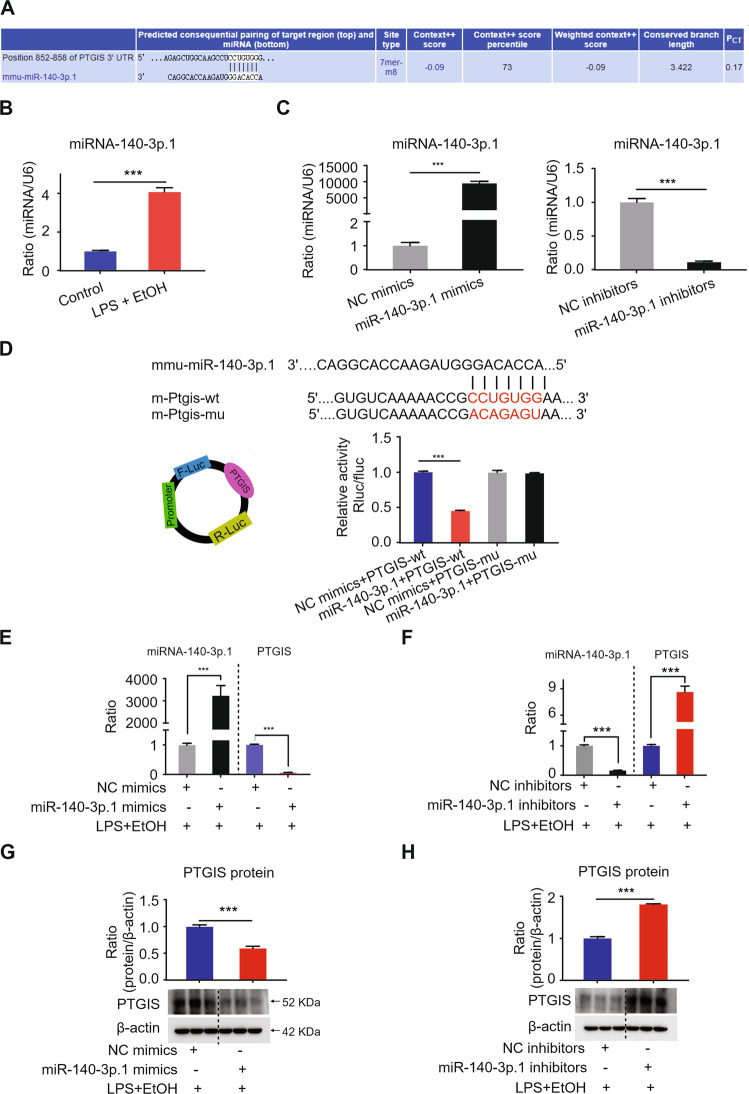
miR-140-3p.1 modulates the expression PTGIS. **A** The PTGIS mRNA targeted miRNA was predicted by TargetScan (http://www.targetscan.org/vert_72/). **B** The expression of miRNA-140-3p.1 in RAW264.7 cells were measured by RT-qPCR. **C** The transfection efficiency of miRNA-140-3p.1 mimics and inhibitors. **D** The predicted miR-140-3p.1 binding site in PTGIS (PTGIS-wt) and the designed mutant sequence (PTGIS-mu) are illustrated. Luciferase reporter assay of HEK 293 T cells co-transfected with PTGIS-wt or PTGIS-mu and miR-140-3p.1 mimics or NC-mimics. **E** miR-140-3p.1 mimic can elevate the expression of miR-140-3p.1 and inhibit PTGIS mRNA levels. **F** miR-140-3p.1 inhibitor can decrease the expression of miR-140-3p.1 and elevated PTGIS mRNA levels. **G** miR-140-3p.1 mimic can inhibit the expression of PTGIS protein. **H** miR-140-3p.1 inhibitor elevated PTGIS protein expression. Values represent the mean ± s.e.m. ^***^p < 0.001 as indicated.

Bioinformatic tools (TargetScan) were used to screen for differentially expressed miRNAs targeting PTGIS. As shown in Fig. 3A, miR-140-3p.1 was the only one conserved and was found to be related to PTGIS expression. Next, we determined the expression of miR-140-3p.1 in the LPS- and EtOH-treated RAW264.7 cells via RT-qPCR. As shown in Fig. 3B, miR-140-3p.1 expression was substantially increased in the LPS- and EtOH-treated group. The transfection efficiency of miR-140-3p.1 mimics and inhibitors were shown in Fig. 3C. We further explored the effect of miR-140-3p.1 on alcohol-induced inflammation in RAW264.7 cells cultured with EtOH and LPS. As shown in Fig. S2A, miR-140-3p.1 mimics promoted M1 polarization and inhibited M2 phenotype, whereas miR-140-3p.1 inhibitors promoted M2 marker expression and inhibited M1 polarization (Fig. S2B). TargetScan showed that the 3′UTR of PTGIS included a putative conserved target site for miR-140-3p.1, and the binding site was conserved (Fig. 3D). Furthermore, miR-140-3p.1 decreased wild-type PTGIS 3′UTR luciferase activity, and the reduction was not observed in a PTGIS 3′UTR reporter, which had mutations in the miR-140-3p.1-binding site (Fig. 3D). In addition, we observed that miR-140-3p.1 mimics significantly decreased PTGIS expression, while miR-140-3p.1 inhibitors markedly elevated PTGIS expression at protein and mRNA levels in RAW264.7 cells cultured with LPS and EtOH (Fig. 3E–H). These results suggest that miR-140-3p.1 is upregulated in LPS- and EtOH-treated RAW264.7 cells and could regulate PTGIS expression.

### The influence of altered PTGIS expression on M1-polarized macrophages

**Fig. 4:**
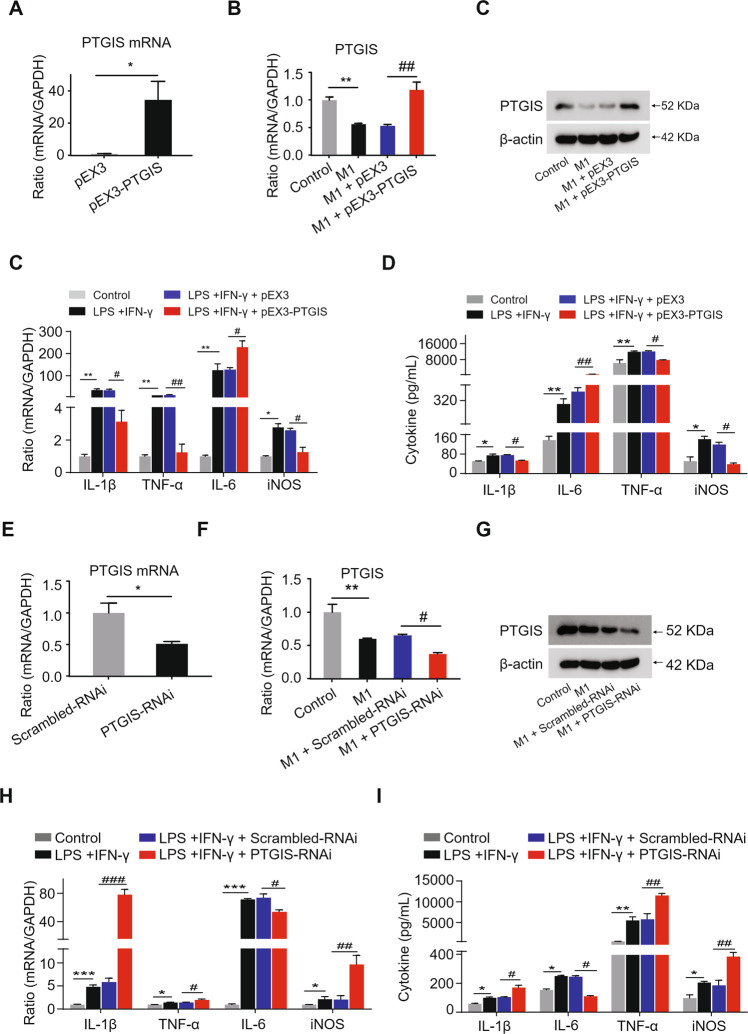
Influence of altered PTGIS expression on M1 polarization. **A** Transfection efficiency of PTGIS overexpression plasmid. **B** The mRNA levels of PTGIS in M1 polarized RAW264.7 cells transfected with PTGIS overexpression plasmid were measured. **C** The mRNA expression of M1 markers. **D** Serum cytokine expression of M1 markers in PTGIS overexpression group. **E** Transfection efficiency of PTGIS-RNAi. **F** The PTGIS mRNA expression in M1 polarized macrophage transfected with PTGIS-RNAi. **G** The expression of PTGIS in M1 polarized RAW264.7 cells transfected with PTGIS-RNAi. **H** mRNA levels of M1 markers. **I** Serum cytokines of M1 markers in PTGIS silencing group. Data represent the mean ± s.e.m. ^*^p < 0.05, ^**^p < 0.01, ^***^p < 0.001, ^#^*p* < 0.05, ^##^*p* < 0.01, ^###^*p* < 0.001 as indicated.

To investigate the influence of PTGIS on M1 polarization, we performed loss- and gain-of-function-experiments. The transfection efficiency of PTGIS plasmid and PTGIS-siRNA is shown in Fig. 4A and E. As shown in Fig. 4B and C, PTGIS overexpression plasmid significantly elevated PTGIS expression at mRNA and protein levels in M1-polarized RAW264.7 cells. Forced PTGIS expression inhibited inflammatory gene expression in M1-polarized macrophages (Fig. 4C and D). As shown in Fig. 4F and G, PTGIS-siRNA inhibited PTGIS expression at the mRNA and protein levels in M1-polarized macrophages. Furthermore, PTGIS silencing induced the elevation of inflammatory gene expression in M1-polarized macrophages (Fig. 4H–I). Interestingly, IL-6 levels decreased in the PTGIS overexpression group and increased in the PTGIS silencing group in M1-polarized macrophages (Fig. 4C, D, H, I). IL-6 acts as a pro-inflammatory cytokine in various conditions [32]. However, Miller et al. demonstrated that the activation of IL-6/STAT3 has a hepatoprotective effect in IL-10^−/−^ mice [33]. Forced PTGIS expression in RAW 264.7 cells without LPS and IFNγ or IL-4 treatment inhibited macrophage M1 polarization and promoted M2 polarization (Fig. S3A), whereas PTGIS silencing in RAW264.7 cells without LPS and IFNγ or IL-4 treatment promoted M1 polarization but inhibited M2 phenotype (Fig. S3B). These results illustrated that forced PTGIS expression inhibits M1 and promotes M2 polarization as well as enhances the hepatoprotective cytokine IL-6 expression.

### The effect of altered PTGIS expression on M2-polarized macrophages

**Fig. 5:**
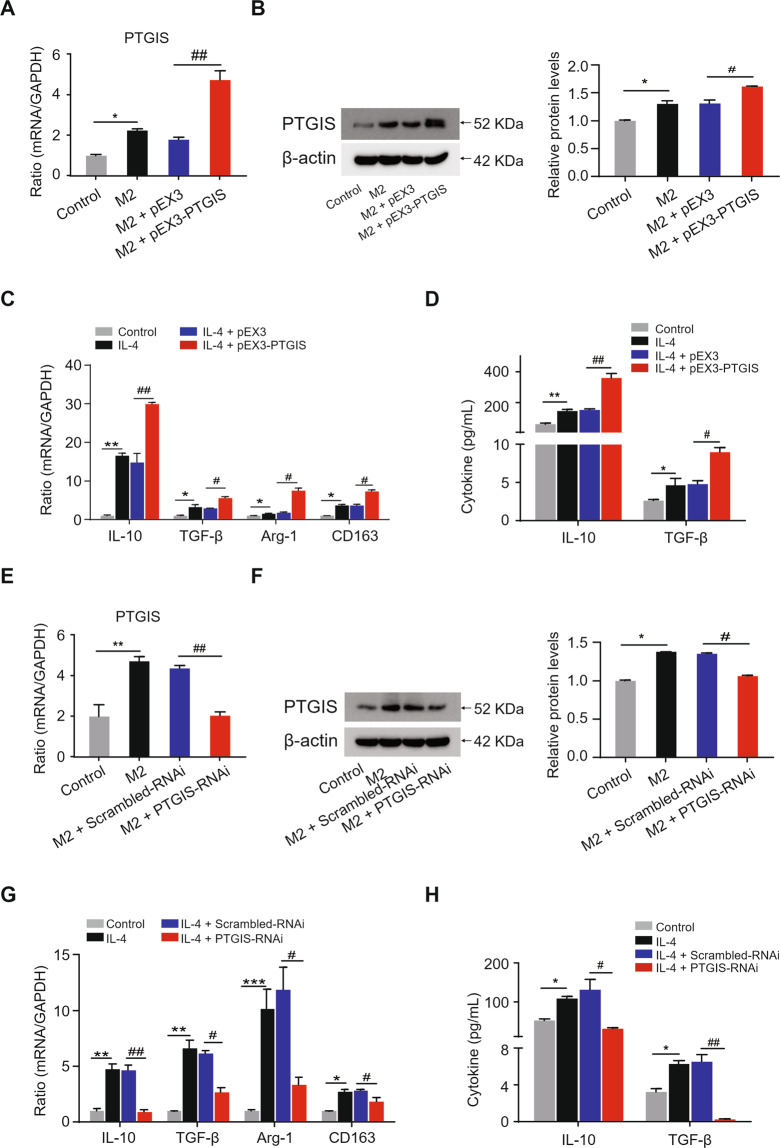
The influence of alternative PTGIS expression on M2 polarization. **A** The mRNA levels of PTGIS in M2 polarized RAW264.7 cells transfected with PTGIS overexpression plasmid. **B** The protein levels of PTGIS in M2 polarized macrophages. **C** The mRNA levels of M2 markers in PTGIS overexpression group. **D** Serum cytokine of M2 markers were detected by ELISA. **E** PTGIS mRNA expression in M2 polarized RAW264.7 cells transfected with PTGIS-RNAi were measured by RT-qPCR. **F** The protein levels of PTGIS were detected. **G** The mRNA levels of M2 markers in PTGIS silencing group. **H** Serum cytokines of M2 markers were detected by ELISA. Values represent the mean ± s.e.m. ^*^p < 0.05, ^**^p < 0.01, ^***^p < 0.001, ^#^*p* < 0.05, ^##^*p* < 0.01, ^###^*p* < 0.001 as indicated.

To investigate the influence of PTGIS on M2 polarization, we performed loss- and gain-of-function-experiments. The PTGIS plasmid significantly increased PTGIS expression at the mRNA and protein levels in M2-polarized macrophages (Fig. 5A, B), and forced PTGIS expression significantly elevated anti-inflammatory gene expression (Fig. 5C, D). Furthermore, PTGIS-siRNA significantly inhibited PTGIS expression in M2-polarized RAW264.7 cells (Fig. 5E, F), and PTGIS silencing induced decreased expression of anti-inflammatory genes (Fig. 5G, H). These results indicated that PTGIS could promote M2 macrophage polarization.

### PTGIS may regulate macrophage polarization via the JAK/STAT pathway

**Fig. 6:**
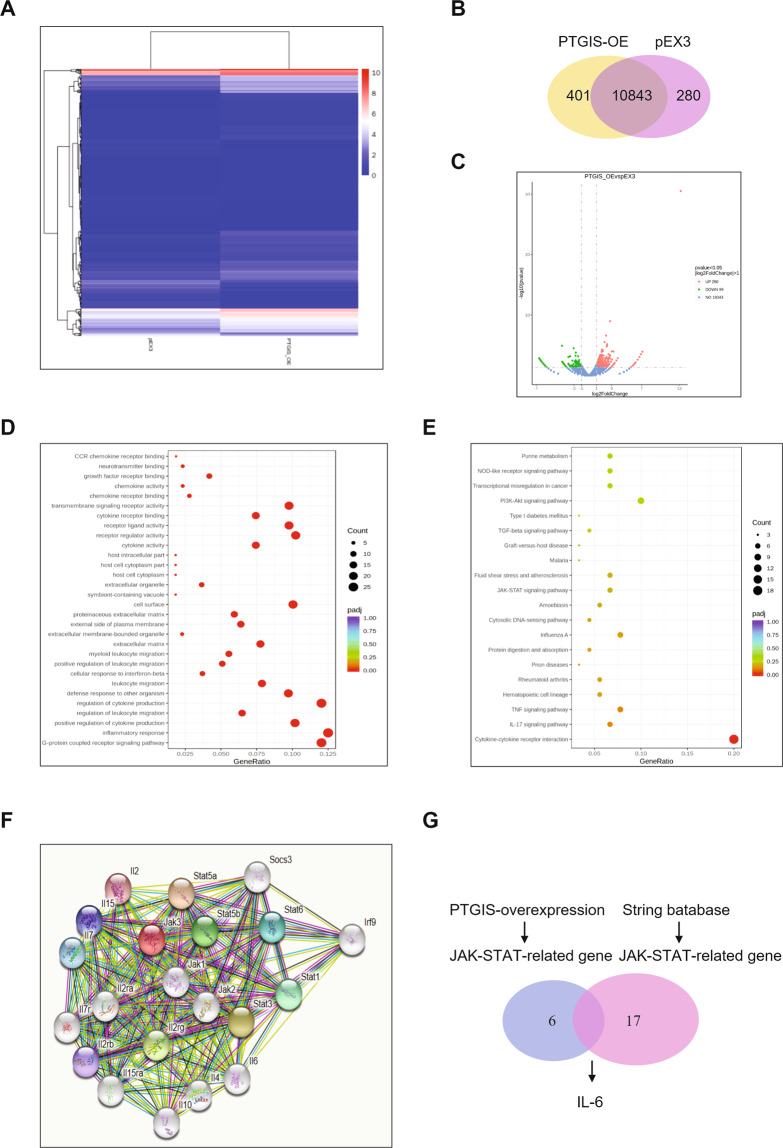
Expression profiles of mRNAs on pEX3 and pEX3-PTGIS overexpressed plasmid transfected RAW264.7 cells. **A** RNA-seq was performed with RNA extracts from RAW264.7 cells transfected with pEX3 or pEX3-PTGIS overexpressed plasmid. Hierarchical cluster analysis of significantly differentially expressed mRNAs: bright blue, under-expression; white, no change; bright red, over-expression. **B** Veen diagram showed mRNAs detected in pEX3 and PTGIS overexpression plasmid transfected group. **C** Volcano plot was presented. **D**, **E** GO and KEGG analysis of altered mRNAs in pEX3 and PTGIS overexpressed transfected group. **F** The proteins interacted with JAK were predicted by STRING database. **G** The overlap between RNA-seq and STRING was IL-6.

To further evaluate the mechanism of PTGIS in regulating macrophage polarization, we forced PTGIS expression in RAW264.7 cells cultured with LPS and EtOH and subsequently isolated total mRNA for RNA-seq (Fig. 6A). As shown in Fig. 6B, we detected 11,244 genes in the PTGIS overexpression group and 11,123 genes in the pEX3-transfected group. As shown in the volcano plot, 290 mRNAs were upregulated and 99 mRNAs were downregulated in the RAW264.7 cells transfected with the PTGIS plasmid compared to those of the pEX3-transfected group (|log2FC | > 1, *p* < 0.05) (Fig. 6C). Gene ontology analysis revealed that PTGIS overexpression affected many genes associated with inflammatory responses, chemokine activity, chemokine receptor activity, and cytokine activity and so on (Fig. 6D). KEGG pathway analysis revealed that forced PTGIS expression could alter JAK-STAT signaling, NOD-like receptor signaling, PI3K-AKT signaling, cytokine-cytokine receptor interaction, IL-17 signaling, etc. (Fig. 6E). As the JAK/STAT pathway plays an important role in macrophage polarization, we focused on the influence of forced PTGIS expression on the JAK/STAT pathway. The proteins interacting with JAK were predicted by the STRING database (Fig. 6F). The overlap between RNA-seq and the STRING database was IL-6 (Fig. 6G). Furthermore, we assessed the effects of altered PTGIS expression on JAK/STAT signaling via western blotting analysis (Fig. S4A–S4D). The results revealed that forced PTGIS expression inhibited JAK/STAT1 activation in M1-polarized macrophages and promoted JAK/STAT6 activation in M2-polarized macrophages (Fig. S4A and S4C). PTGIS silencing promoted JAK/STAT1 activation in M1-polarized macrophages and inhibited JAK/STAT6 activation in M2-polarized macrophages (Fig. S4B and S4D). These results suggest that PTGIS might regulate macrophage polarization through JAK/STAT signaling.

## Discussion

**Fig. 7:**
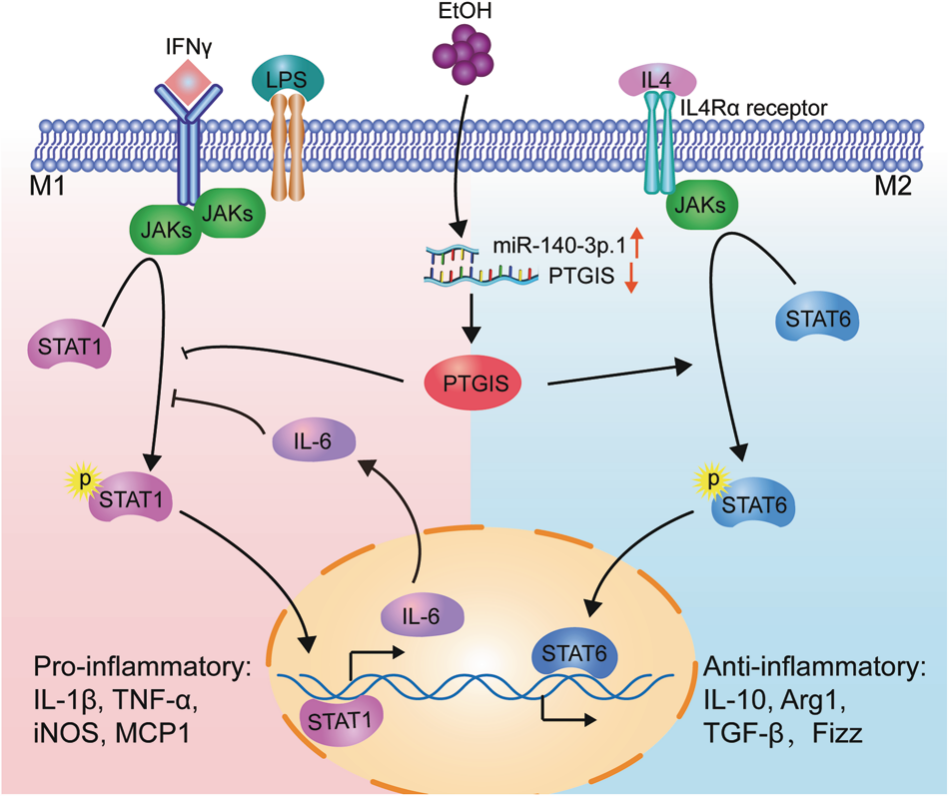
Schematic presentation of the effects of PTGIS on macrophage polarization on alcohol-induces liver injury. Ethanol and LPS induced miR-140-3p.1 expression. Subsequently, miR-140-3p.1 inhibited PTGIS expression. Overexpression of PTGIS in vivo and in vitro inhibits macrophage M1 polarization and promotes M2 polarization through JAK-STAT signaling.

Alcohol consumption is a leading risk factor for death and disability worldwide. A systematic analysis of the global burden of alcohol revealed that the level of consumption that minimizes heath loss is zero [1]. ALD appears as a wide range of disorders, ranging from simple liver steatosis to more severe forms of liver injury, including alcoholic hepatitis, liver fibrosis, cirrhosis, and hepatocellular carcinoma [33]. Ethanol metabolism-associated oxidative stress, glutathione depletion, abnormal methionine metabolism, malnutrition, ethanol-mediated induction of gut endotoxin leakage, and subsequent activation of macrophages play important roles in the pathogenesis of ALD [34–39]. In this study, we illustrated for the first time, to our knowledge, that PTGIS decreased in a chronic alcohol-consuming mouse model, and overexpression of PTGIS in vivo alleviated liver steatosis, inflammatory cell infiltration, and macrophage switch to the M1 phenotype (Fig. 7).

Ethanol increases fatty acid synthesis in hepatocytes via the upregulation of sterol regulatory element-binding protein 1c [40], a transcription factor that promotes fatty acid synthesis via the upregulation of lipogenic genes and the inhibition of PPARα [41, 42], a nuclear hormone receptor that controls the transcription of a range of genes involved in free fatty acid transport and oxidation. In addition, ethanol can affect the activities of enzymes involved in fat metabolism by inhibiting AMP-activated protein kinase, which reduces fat metabolism and fatty liver [43, 44]. In this study, we demonstrated that PTGIS overexpression in vivo alleviated fatty acid accumulation in the liver, as determined by Oil Red O staining (Figs. 1A and 2C).

Alcohol consumption not only causes enteric dysbiosis and bacterial over-growth [45] but also increases gut permeability and the translocation of bacteria-derived LPS from the gut to the liver [46]. LPS interacts with Toll-like receptor 4 (TLR4) to activate the MyD88-independent signaling pathway, leading to the production of proinflammatory cytokines, including IL-1β and TNF-α [47–49]. Interestingly, activation of TLR4 and complement factors also causes macrophages to produce hepatoprotective cytokines, such as IL-6, and anti-inflammatory cytokines, such as IL-10 [33, 50]. In this study, the results indicated that chronic alcohol consumption induces M1- and M2-polarized macrophages to increase simultaneously (Fig. 1E–G) and inhibit PTGIS expression in vivo and in vitro (Fig. 1H–K). Overexpression of PTGIS could alleviate liver injury in vivo (Figs. 2C, S1B, and S1C). Forced PTGIS expression inhibited macrophage M1 polarization and promoted M2 polarization both in vivo and in vitro (Figs. 2D–F, 4, and 5). A range of evidence demonstrated that the JAK/STATs signaling pathway plays a pivotal role in regulating macrophage polarization. RNA-seq analysis revealed that that the genes regulated by PTGIS were enriched in the JAK/STAT pathway (Fig. 6D and E). Forced PTGIS expression inhibited JAK/STAT1 activation in M1-polarized macrophages and promoted JAK/STAT6 activation in M2-polarized macrophages (Fig. S4A and S4C). PTGIS silencing promoted JAK/STAT1 activation in M1-polarized macrophages and inhibited JAK/STAT6 activation in M2-polarized macrophages (Fig. S4B and S4D). The overlap between the altered genes associated with PTGIS and the genes interacted with JAKs was IL-6 (Fig. 6G). The activation of STAT1 (pSTAT1) and STAT3 (pSTAT3) plays a pivotal role in controlling liver steatosis and liver inflammation [51]. STAT3, which is activated by IL-6 plays an important role in hepatoprotection, liver regeneration, and glucose homeostasis by inducing a variety of anti-apoptotic and mitogenic proteins [51]. Miller et al. demonstrated that deletion of IL-6 or hepatic STAT3 increased STAT1 activation in HFD-fed IL-10^−/−^ mice [33]. IL-10 is a well-documented anti-inflammatory cytokine and IL-6 is a pro-inflammatory cytokine [52]. However, IL-6 can act as an anti-inflammatory cytokine by inhibiting the activation of STAT1 [33]. Therefore, we hypothesized that chronic alcohol consumption leads to liver injury and inflammation and forced PTGIS expression can inhibit M1 macrophage polarization by inhibiting the activation of STAT1 through IL-6, and IL-6 acts as an anti-inflammatory cytokine on this occasion.

A range of evidence over the last decade indicates a pivotal role of miRNAs as key mediators of macrophage differentiation, infiltration, and activation. Initial studies have indicated the property of miRNAs to regulate the magnitude of the innate response, participating as integral components of feedback loop regulatory mechanisms, which significantly shape the inflammatory response [53]. In addition, recent studies have demonstrated that miRNAs regulate macrophage differentiation and polarization [54]. In short, a complex and highly regulated network of miRNAs regulating inflammatory pathways by targeting components of TLR signaling and influencing downstream inflammatory cytokine production has been defined. In this study, we demonstrated that the regulation of PTGIS in macrophage polarization could be influenced by miRNA-140-3p.1 (Fig. 3).

Macrophages are hindered by their versatility, and can adapt their phenotype (to become pro-inflammatory or anti-inflammatory) in response to molecular signals in healthy or injured livers. Historically, the plasticity of macrophages has been described using the terms classically activated (M1 type) and alternatively activated (M2 type). However, this nomenclature is too simple to describe the polarization phenotypes of macrophages driven by many different stimuli in the microenvironment. Ly-6C is a cell surface glycoprotein widely used to identify functionally discrete murine circulating monocyte populations: Ly-6C^hi^ monocytes and Ly-6C^lo^. Ly-6C^hi^ monocytes are recruited early to inflammatory environments and are thought to be proinflammatory, whereas Ly-6C^lo^ monocytes are a patrolling cell type and can supplement resident tissue macrophages [55, 56]. Further studies are needed to investigate the influence of altered PTGIS expression on differential Ly-6C expression in chronicALD.

In conclusion, as shown in Fig. 7, we demonstrated that PTGIS expression was downregulated in chronic alcohol-consuming mice via elevated miR-140-3p.1 expression. Forced PTGIS expression in vivo and in vitro can inhibit macrophage switch to the M1 phenotype and promote M2 polarization. RNA-seq results showed that PTGIS might regulate macrophage polarization through the JAK/STAT pathway, and IL-6 might be involved in inhibiting the activation of STAT1. This study revealed that PTGIS might be a therapeutic target for ALD.

## Supplementary information

Supplementary materials

## Data Availability

The datasets used and analyzed in this study are available from the corresponding author on reasonable request.

## References

[1] Griswold MG, Fullman N, Hawley C, Arian N, Zimsen SRM, Tymeson HD, et al. Alcohol use and burden for 195 countries and territories, 1990-2016: a systematic analysis for the Global Burden of Disease Study 2016. Lancet. 2018;392:1015-35. 10.1016/S0140-6736(18)31310-2 PMC6148333 30146330

[2] Liangpunsakul S, Haber P, McCaughan GW (2016). Alcoholic liver disease in Asia, Europe, and North America. Gastroenterology.

[3] Zhou Z, Zhong W (2017). Targeting the gut barrier for the treatment of alcoholic liver disease. Liver Res.

[4] Yang AM, Inamine T, Hochrath K, Chen P, Wang L, Llorente C (2017). Intestinal fungi contribute to development of alcoholic liver disease. J Clin Invest.

[5] Mosser DM, Edwards JP (2008). Exploring the full spectrum of macrophage activation. Nat Rev Immunol.

[6] Gordon S, Martinez FO (2010). Alternative activation of macrophages: mechanism and functions. Immunity.

[7] Biswas SK, Mantovani A (2010). Macrophage plasticity and interaction with lymphocyte subsets: cancer as a paradigm. Nat Immunol.

[8] Biswas SK, Mantovani A (2012). Orchestration of metabolism by macrophages. Cell Metab.

[9] Wan J, Benkdane M, Teixeira-Clerc F, Bonnafous S, Louvet A, Lafdil F (2014). M2 Kupffer cells promote M1 Kupffer cell apoptosis: a protective mechanism against alcoholic and nonalcoholic fatty liver disease. Hepatology.

[10] Darnell JE, Kerr IM, Stark GR (1994). Jak-STAT pathways and transcriptional activation in response to IFNs and other extracellular signaling proteins. Science.

[11] Takeda K, Tanaka T, Shi W, Matsumoto M, Minami M, Kashiwamura S (1996). Essential role of Stat6 in IL-4 signalling. Nature.

[12] Martinez FO, Helming L, Gordon S (2009). Alternative activation of macrophages: an immunologic functional perspective. Annu Rev Immunol.

[13] Fruman DA, Snapper SB, Yballe CM, Davidson L, Yu JY, Alt FW (1999). Impaired B cell development and proliferation in absence of phosphoinositide 3-kinase p85alpha. Science.

[14] Ricote M, Li AC, Willson TM, Kelly CJ, Glass CK (1998). The peroxisome proliferator-activated receptor-gamma is a negative regulator of macrophage activation. Nature.

[15] Louvet A, Teixeira-Clerc F, Chobert MN, Deveaux V, Pavoine C, Zimmer A (2011). Cannabinoid CB2 receptors protect against alcoholic liver disease by regulating Kupffer cell polarization in mice. Hepatology.

[16] Yang Y, Wu XQ, Li WX, Huang HM, Li HD, Pan XY (2018). PSTPIP2 connects DNA methylation to macrophage polarization in CCL4-induced mouse model of hepatic fibrosis. Oncogene.

[17] Ebert MS, Sharp PA (2012). Roles for microRNAs in conferring robustness to biological processes. Cell.

[18] Curtale G, Renzi TA, Mirolo M, Drufuca L, Albanese M, De Luca M (2018). Multi-step regulation of the TLR4 pathway by the miR-125a~99b~let-7e cluster. Front Immunol.

[19] Rossato M, Curtale G, Tamassia N, Castellucci M, Mori L, Gasperini S (2012). IL-10-induced microRNA-187 negatively regulates TNF-alpha, IL-6, and IL-12p40 production in TLR4-stimulated monocytes. Proc Natl Acad Sci USA.

[20] Curtale G, Mirolo M, Renzi TA, Rossato M, Bazzoni F, Locati M (2013). Negative regulation of Toll-like receptor 4 signaling by IL-10-dependent microRNA-146b. Proc Natl Acad Sci USA.

[21] Smith WL, Urade Y, Jakobsson PJ (2011). Enzymes of the cyclooxygenase pathways of prostanoid biosynthesis. Chem Rev.

[22] Cebola I, Peinado MA (2012). Epigenetic deregulation of the COX pathway in cancer. Prog Lipid Res.

[23] Frigola J, Munoz M, Clark SJ, Moreno V, Capella G, Peinado MA (2005). Hypermethylation of the prostacyclin synthase (PTGIS) promoter is a frequent event in colorectal cancer and associated with aneuploidy. Oncogene.

[24] Keith RL, Miller YE, Hoshikawa Y, Moore MD, Gesell TL, Gao B (2002). Manipulation of pulmonary prostacyclin synthase expression prevents murine lung cancer. Cancer Res.

[25] Keith RL, Miller YE, Hudish TM, Girod CE, Sotto-Santiago S, Franklin WA (2004). Pulmonary prostacyclin synthase overexpression chemoprevents tobacco smoke lung carcinogenesis in mice. Cancer Res.

[26] Pradono P, Tazawa R, Maemondo M, Tanaka M, Usui K, Saijo Y (2002). Gene transfer of thromboxane A(2) synthase and prostaglandin I(2) synthase antithetically altered tumor angiogenesis and tumor growth. Cancer Res.

[27] Cutler NS, Graves-Deal R, LaFleur BJ, Gao Z, Boman BM, Whitehead RH (2003). Stromal production of prostacyclin confers an antiapoptotic effect to colonic epithelial cells. Cancer Res.

[28] Cebola I, Custodio J, Munoz M, Diez-Villanueva A, Pare L, Prieto P (2015). Epigenetics override pro-inflammatory PTGS transcriptomic signature towards selective hyperactivation of PGE2 in colorectal cancer. Clin Epigenetics.

[29] Hargreaves DC, Horng T, Medzhitov R (2009). Control of inducible gene expression by signal-dependent transcriptional elongation. Cell.

[30] Bertola A, Mathews S, Ki SH, Wang H, Gao B (2013). Mouse model of chronic and binge ethanol feeding (the NIAAA model). Nat Protoc.

[31] Holt MP, Cheng L, Ju C (2008). Identification and characterization of infiltrating macrophages in acetaminophen-induced liver injury. J Leukoc Biol.

[32] Ishihara K, Hirano T (2002). IL-6 in autoimmune disease and chronic inflammatory proliferative disease. Cytokine Growth Factor Rev.

[33] Miller AM, Wang H, Bertola A, Park O, Horiguchi N, Ki SH (2011). Inflammation-associated interleukin-6/signal transducer and activator of transcription 3 activation ameliorates alcoholic and nonalcoholic fatty liver diseases in interleukin-10-deficient mice. Hepatology.

[34] Lieber CS (1994). Susceptibility to alcohol-related liver injury. Alcohol Alcohol Suppl.

[35] Thurman RG, Bradford BU, Iimuro Y, Knecht KT, Arteel GE, Yin M (1998). The role of gut-derived bacterial toxins and free radicals in alcohol-induced liver injury. J Gastroenterol Hepatol.

[36] Tsukamoto H, Lu SC (2001). Current concepts in the pathogenesis of alcoholic liver injury. FASEB J.

[37] Hoek JB, Cahill A, Pastorino JG (2002). Alcohol and mitochondria: a dysfunctional relationship. Gastroenterology.

[38] Lumeng L, Crabb DW (2001). Alcoholic liver disease. Curr Opin Gastroenterol.

[39] Arteel G, Marsano L, Mendez C, Bentley F, McClain CJ (2003). Advances in alcoholic liver disease. Best Pract Res Clin Gastroenterol.

[40] You M, Fischer M, Deeg MA, Crabb DW (2002). Ethanol induces fatty acid synthesis pathways by activation of sterol regulatory element-binding protein (SREBP). J Biol Chem.

[41] Yu S, Rao S, Reddy JK (2003). Peroxisome proliferator-activated receptors, fatty acid oxidation, steatohepatitis and hepatocarcinogenesis. Curr Mol Med.

[42] Wagner M, Zollner G, Trauner M (2011). Nuclear receptors in liver disease. Hepatology.

[43] Viollet B, Guigas B, Leclerc J, Hebrard S, Lantier L, Mounier R (2009). AMP-activated protein kinase in the regulation of hepatic energy metabolism: from physiology to therapeutic perspectives. Acta Physiol (Oxf).

[44] Li Y, Xu S, Mihaylova MM, Zheng B, Hou X, Jiang B (2011). AMPK phosphorylates and inhibits SREBP activity to attenuate hepatic steatosis and atherosclerosis in diet-induced insulin-resistant mice. Cell Metab.

[45] Yan AW, Fouts DE, Brandl J, Starkel P, Torralba M, Schott E (2011). Enteric dysbiosis associated with a mouse model of alcoholic liver disease. Hepatology.

[46] Rao R (2009). Endotoxemia and gut barrier dysfunction in alcoholic liver disease. Hepatology.

[47] Pritchard MT, McMullen MR, Stavitsky AB, Cohen JI, Lin F, Edward Medof M (2007). Differential contributions of C3, C5, and decay-accelerating factor to ethanol-induced fatty liver in mice. Gastroenterology.

[48] Cohen JI, Roychowdhury S, McMullen MR, Stavitsky AB, Nagy LE (2010). Complement and alcoholic liver disease: role of C1q in the pathogenesis of ethanol-induced liver injury in mice. Gastroenterology.

[49] Ding WX, Li M, Chen X, Ni HM, Lin CW, Gao W (2010). Autophagy reduces acute ethanol-induced hepatotoxicity and steatosis in mice. Gastroenterology.

[50] Horiguchi N, Wang L, Mukhopadhyay P, Park O, Jeong WI, Lafdil F (2008). Cell type-dependent pro- and anti-inflammatory role of signal transducer and activator of transcription 3 in alcoholic liver injury. Gastroenterology.

[51] Gao B (2005). Cytokines, STATs and liver disease. Cell Mol Immunol.

[52] He G, Karin M (2011). NF-kappaB and STAT3 - key players in liver inflammation and cancer. Cell Res.

[53] Mehta A, Baltimore D (2016). MicroRNAs as regulatory elements in immune system logic. Nat Rev Immunol.

[54] Su X, Yu Y, Zhong Y, Giannopoulou EG, Hu X, Liu H (2015). Interferon-gamma regulates cellular metabolism and mRNA translation to potentiate macrophage activation. Nat Immunol.

[55] Gordon S, Taylor PR (2005). Monocyte and macrophage heterogeneity. Nat Rev Immunol.

[56] Ingersoll MA, Spanbroek R, Lottaz C, Gautier EL, Frankenberger M, Hoffmann R (2010). Comparison of gene expression profiles between human and mouse monocyte subsets. Blood.

