# Supplementary material for: Alternative activation of macrophages by prostacyclin synthase ameliorates alcohol induced liver injury
Source: Lab Invest. 2021 Jun 10;101(9):1210–24. doi: 10.1038/s41374-021-00531-7 (PMC8367821; doi:10.1038/s41374-021-00531-7)
Supplement: Supplementary file 1 — Supplementary materials [file 41374_2021_531_MOESM1_ESM.docx]

**Supplementary materials:**

**Figure S1.**


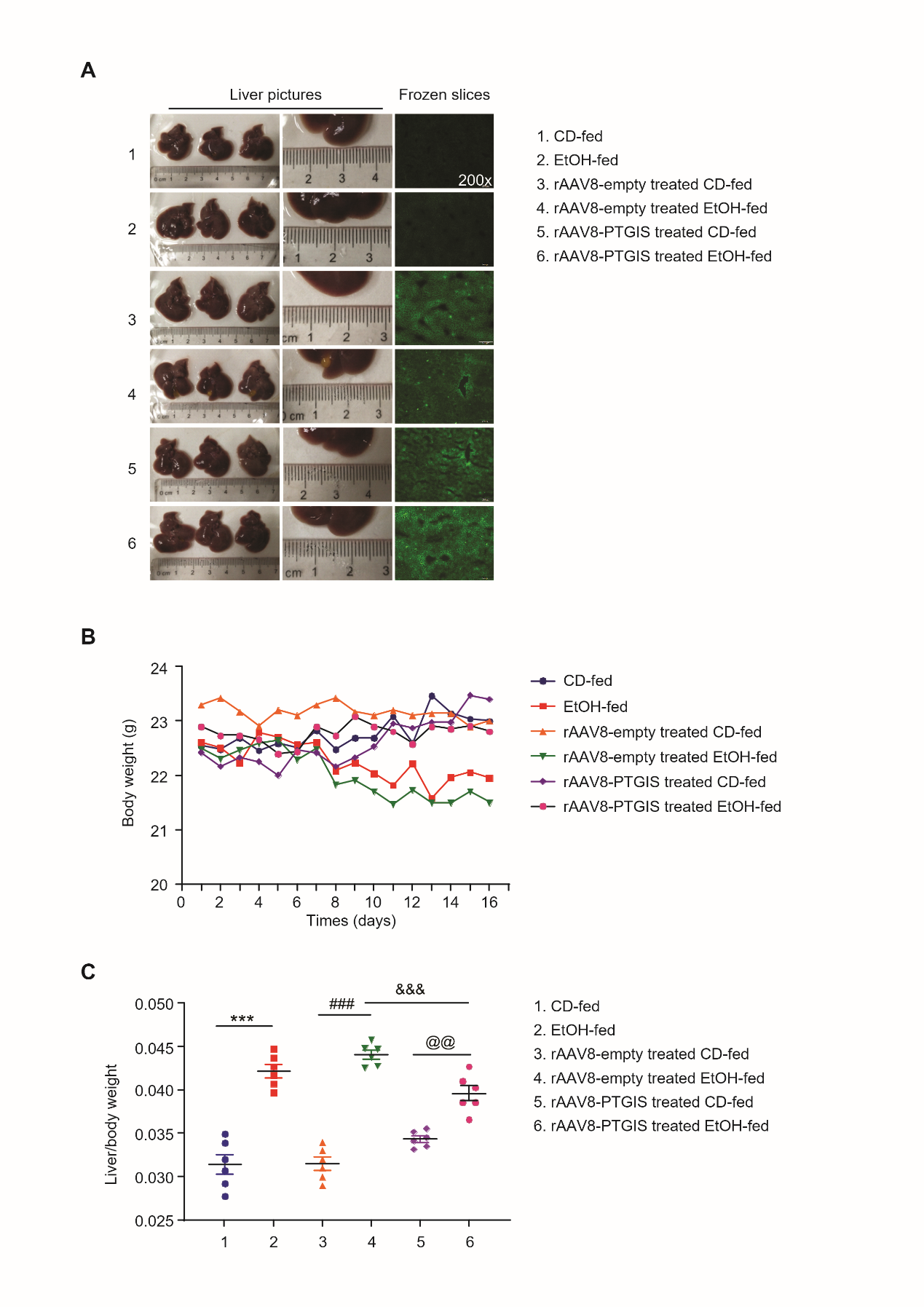


**Figure S2.**


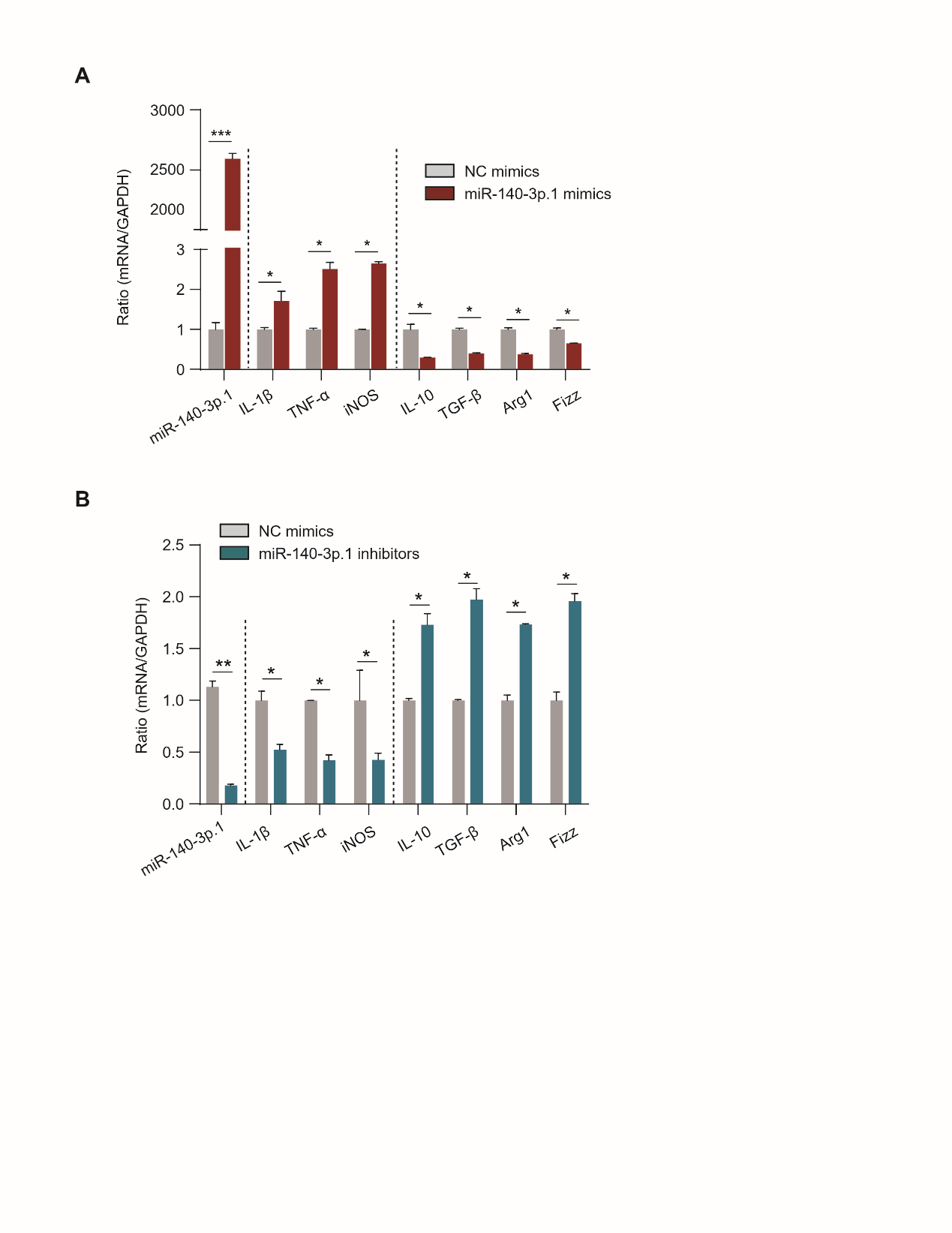


**Figure S3.**


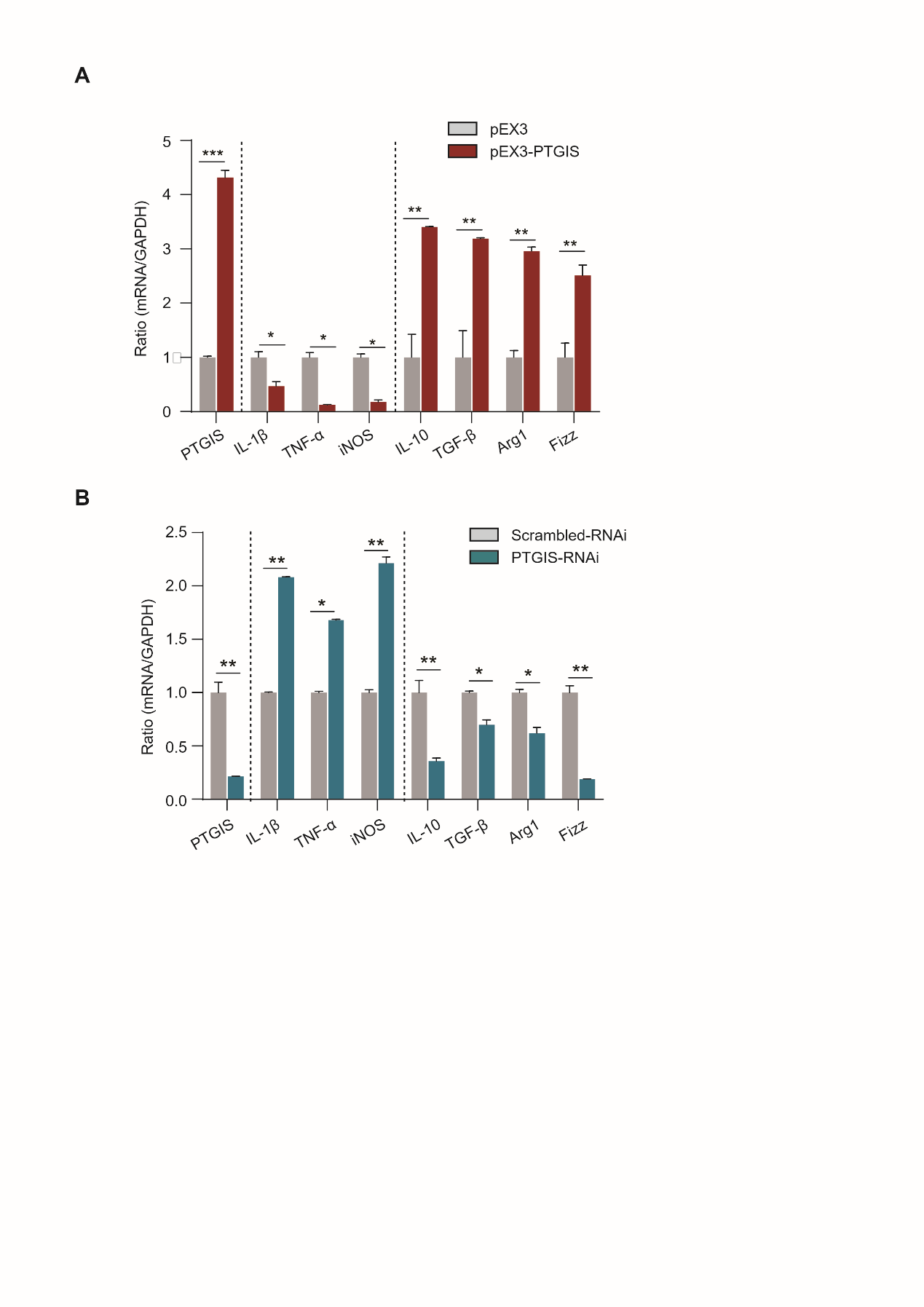


**Figure S4.**


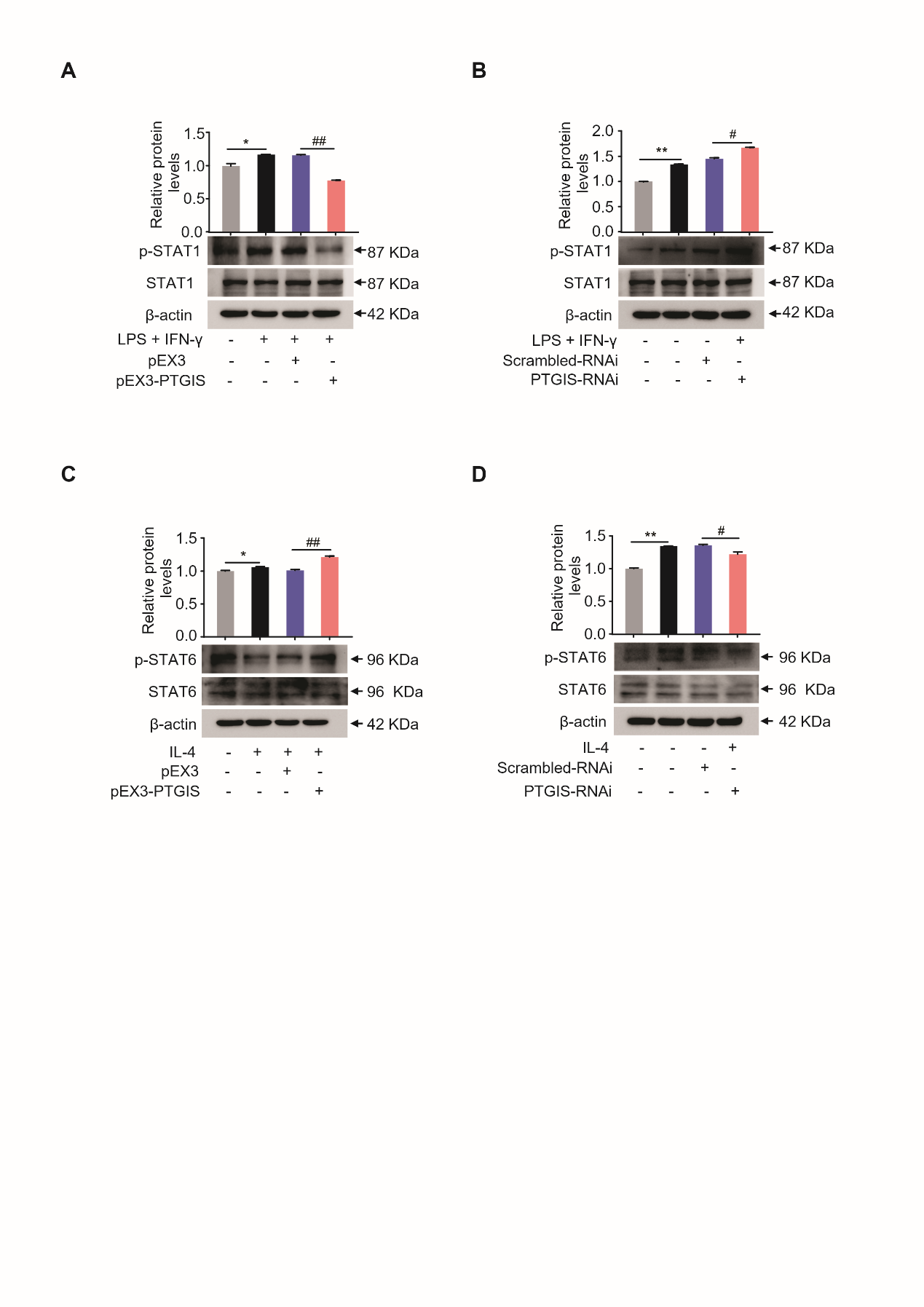


**Supplementary Figure legends:**

**Figure S1. Influence of PRGIS overexpression *in vivo*.** **(A)** Gross pathology of liver tissues. Representative pictures of frozen slices were presented (Original magnification was 200x). **(B)** Body weight were shown. **(C)** The ratio of liver-to-body weight.

**Figure S2. Function of miR-140-3p.1 in EtOH-induced liver injury.** The influence of miR-140-3p.1 mimics **(A)** and inhibitors **(B)** on macrophage polarization.

**Figure S3. The influence of altered PTGIS expression on macrophage polarization.** The functional role of PTGIS overexpression **(A)** and PTGIS silencing **(B)** on macrophage polarization in RAW264.7 cell at basal levels (without IL-4 or LPS treatment).

**Figure S4. The functional role of loss- and gain-expression of PTGIS on JAK/STAT signaling.** **(A)** and **(B)** The influence of PTGIS overexpression or silence on the expression of STAT1 and p-STAT1 in M1 polarized RAW264.7 cells. **(C)** and **(D)** The effect of PTGIS overexpression or silence on the expression of STAT6 and p-STAT6 expression in M2 polarized RAW264.7 cells. Values represent the mean ± s.e.m. ^*^*p* <0.05, ^**^*p* <0.01, ^#^*p* <0.05, ^##^*p* <0.01 as indicated.
